# Generation of the First Human In Vitro Model for McArdle Disease Based on iPSC Technology

**DOI:** 10.3390/ijms232213964

**Published:** 2022-11-12

**Authors:** María del Carmen Ortuño-Costela, Victoria Cerrada, Ana Moreno-Izquierdo, Inés García-Consuegra, Camille Laberthonnière, Mégane Delourme, Rafael Garesse, Joaquín Arenas, Carla Fuster García, Gema García García, José María Millán, Frédérique Magdinier, María Esther Gallardo

**Affiliations:** 1 Grupo de Investigación Traslacional con células iPS, Instituto de Investigación Sanitaria Hospital 12 de Octubre (imas12), 28041 Madrid, Spain; 2 Departamento de Bioquímica, Facultad de Medicina, Universidad Autónoma de Madrid, Instituto de Investigaciones Biomédicas “Alberto Sols”, (UAM-CSIC), 28029 Madrid, Spain; 3 Servicio de Genética, Hospital 12 de Octubre, Instituto de Investigación Sanitaria Hospital 12 de Octubre (imas12), 28041 Madrid, Spain; 4 Laboratorio de Enfermedades Mitocondriales y Neuromusculares, Instituto de Investigación Sanitaria Hospital 12 de Octubre (imas12), 28041 Madrid, Spain; 5 Centro de Investigación Biomédica en Red de Enfermedades Raras (CIBERER), 28029 Madrid, Spain; 6 Aix-Marseille University, INSERM, MMG, 13385 Marseille, France; 7 Grupo de Investigación en Biomedicina Molecular, Celular y Genómica, Instituto de Investigación Sanitaria La Fe (IIS La Fe), 46029 Valencia, Spain

**Keywords:** iPSCs, McArdle disease, *PYGM*, disease modelling, skeletal muscle differentiation, gene editing, CRISPR/Cas9, isogenic control, read-through drugs

## Abstract

McArdle disease is a rare autosomal recessive disorder caused by mutations in the *PYGM* gene. This gene encodes for the skeletal muscle isoform of glycogen phosphorylase (myophosphorylase), the first enzyme in glycogenolysis. Patients with this disorder are unable to obtain energy from their glycogen stored in skeletal muscle, prompting an exercise intolerance. Currently, there is no treatment for this disease, and the lack of suitable in vitro human models has prevented the search for therapies against it. In this article, we have established the first human iPSC-based model for McArdle disease. For the generation of this model, induced pluripotent stem cells (iPSCs) from a patient with McArdle disease (harbouring the homozygous mutation c.148C>T; p.R50* in the *PYGM* gene) were differentiated into myogenic cells able to contract spontaneously in the presence of motor neurons and generate calcium transients, a proof of their maturity and functionality. Additionally, an isogenic skeletal muscle model of McArdle disease was created. As a proof-of-concept, we have tested in this model the rescue of *PYGM* expression by two different read-through compounds (PTC124 and RTC13). The developed model will be very useful as a platform for testing drugs or compounds with potential pharmacological activity.

## 1. Introduction

McArdle disease (also known as glycogen storage disease type V, OMIM 232600) is an autosomal recessive rare disorder caused by mutations in the *PYGM* gene [1]. This gene encodes for the muscle isoform of glycogen phosphorylase (myophosphorylase), which catalyses and regulates glycogen breakdown to glucose-1-phosphate during glycogenolysis in skeletal muscle [2]. To date, more than 150 mutations have been described in the *PYGM* gene associated with McArdle disease, the nonsense mutation c.148C>T; p.R50* being the most prevalent in the Caucasian population [3].

As a consequence of deficient myophosphorylase activity, McArdle disease patients are unable to obtain energy from their muscle glycogen stores and experience exercise intolerance [4]. This translates into acute crisis of fatigue and weakness when exercising, especially at the beginning, accompanied by muscle cramps, myalgia, and contractures. Rhabdomyolysis also occurs often, as reflected by high serum levels of creatine kinase (hyperCKaemia) or myoglobinuria [5], with the risk of developing renal failure [6].

Currently, there is no treatment for the disease. Several pharmacological options, such as branched-chain amino acids, verapamil, and valproic acid, among others, have been tested without showing any promising or conclusive results [7,8]. The only therapy that seems to have some benefits is the recommendation of low to moderate exercise, as physically active patients have demonstrated major exercise tolerance [9].

There are four animal models available for the study of McArdle disease: two spontaneous models in Charolais cattle [10] and Merino sheep [11], a knock-in the mouse model for the *PYGM* p.R50* mutation [12] and a zebrafish model [13]. Although they have improved the knowledge about McArdle disease, they fail to completely mimic the human disorder phenotype [14]. Additionally, none allows the implementation of a high-throughput drug screening. The generation of an in vitro human model becomes imperative to explore the possibility of a pharmacological option for McArdle disease patients.

Since their discovery in 2006 by Dr Yamanaka, induced pluripotent stem cells (iPSCs) have marked a milestone in biomedical research [15,16]. Due to their auto-renewal capacity and potential to virtually differentiate to any cell type of the human body, the applications of iPSCs are innumerable [17]. In the last decade, iPSCs have become an essential tool for understanding embryonic development, modelling diseases, and searching for new treatments [18,19,20], with many on-going clinical trials that underline the significance of this technology [21]. To generate a particular disease model, patient-derived iPSCs must be differentiated to the disease target cell type. Several differentiation protocols have already been developed in the last years to generate skeletal muscle from iPSCs, with diverse strategies. Some protocols are based on the overexpression of selected myogenic transcription factors (like PAX7 or MYOD1) to force iPSCs to differentiate towards a myogenic lineage [22,23], whereas others try to mimic embryonic development by supplementing the iPSCs culture media with different small molecules and defined factors [24]. The selection of the most appropriate differentiation protocol for each application must be performed carefully, considering their main advantages and possible drawbacks [25].

Establishing the most suitable control for a specific disease model is essential to draw the correct conclusions during the evaluation of pathological mechanisms or possible therapies. Genetic variability between individuals is a key factor that should be considered when comparing a “control” versus a pathogenic cell line. Isogenic controls have gained prominence in recent years, representing the finest reference values. A few years ago, the creation of isogenic controls from iPSCs could constitute an obstacle, but nowadays the development of the CRISPR technology has provided the scientific community with an invaluable tool for gene editing [26], allowing the generation of the ideal controls for in vitro disease models in an easy, affordable, and rapid manner.

In this article, the first human iPSC-based model for McArdle disease was generated. For this purpose, iPSCs of a McArdle patient harbouring the homozygous mutation in the *PYGM* gene c.148C>T; p.R50* were successfully differentiated into innervated mature skeletal muscle, able to spontaneously contract. Additionally, using the CRISPR system, we have also created an iPSC-based isogenic model of McArdle disease. This model will be very useful as a platform to perform high-throughput drug screenings to search for a therapy for this disorder. As a proof of concept two read-through drugs (PTC124 and RTC13) were tested using the developed model.

## 2. Results

### 2.1. Establishment of the iPSC Lines C10 and MA4

The iPSC line C10, generated as a control, has the same origin as the previously published line IISHDOi002-A [27]. Likewise, the iPSC line MA4 has the same origin as the previously published iPSC line IISHDOi001-A (named here as MA1) [28], and their evaluation in conjunction would allow avoiding clonal effects in further experiments.

A complete battery of tests was performed to confirm the pluripotency and integrity of the established lines after the reprogramming event. Both stained positive for alkaline phosphatase, as we can observe in Figures S1a and S2b, showing a typical human embryonic stem cell (hESC)-like morphology. The presence of the mutation c.148C>T; p.R50* in the *PYGM* sequence of the MA4 iPSC line was confirmed by Sanger sequencing (Figure S2a). The lines also exhibited positive expression of certain pluripotency markers, analysed by immunofluorescence, such as NANOG, OCT4, and SOX2 as nuclear markers, along with SSEA4, SSEA3, TRA-1-81, and TRA-1-60 as surface markers (Figures S1b and S2d). We also verified the expression of the pluripotency genes *OCT4*, *SOX2*, *CRIPTO*, *NANOG*, and *REX1* by RT-qPCR, as it shows in Figures S1c and S2c. Additionally, we performed an in vitro differentiation assay to demonstrate the functional pluripotency of the lines, finding that they had the ability to differentiate to cells of all three germ layers: endoderm, ectoderm, and mesoderm (Figures S1d and S2e). The lines were mycoplasma free (Figures S1e and S2h) and were demonstrated to have the same origin as the previously published lines IISHDOi002-A and IISHDOi001-A respectively, using a DNA fingerprinting analysis (data available upon request). We also confirmed the clearance of the vectors and the exogenous reprogramming factor genes by RT-PCR (Figures S1f and S2g). The iPSC lines MA4 and C10 exhibited a normal 46, XX and 46, XY karyotype respectively (Figures S1g and S2f).

### 2.2. Differentiation to Innervated Skeletal Muscle Cells

**Figure 1:**
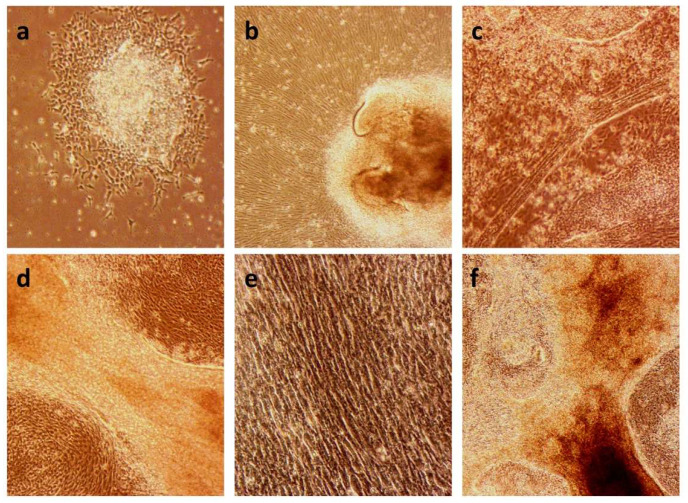
Changes in morphology of the cell cultures throughout the differentiation protocol. (**a**) MA1 line at day 4 of differentiation (10×). We can observe the centre of the clump still maintaining its stemness, whereas the border starts the differentiation process. (**b**) MA1 line at day 10 of differentiation (10×). The cells start to align while expanding from the undifferentiated clump. (**c**) C10 line at day 22 of differentiation (10×). Formation of myofiber-like structures. (**d**) C10 line at day 25 of differentiation (20×). We can observe in detail the structure of the myofibers formed in the differentiated culture. (**e**) C10 line at day 25 of differentiation (20×). Details of the alignment of the cells in the differentiated culture. (**f**) MA4 line at day 28 of differentiation (10×). The culture exhibits a great density, with formed myofiber-like structures.

For the establishment of a McArdle disease model, the next step after the generation of the iPSC lines would be their differentiation towards the cell lineage mainly affected in the disease: skeletal muscle cells. The control iPSC lines C10 and AG09G (previously generated by Dr Magdinier’s group), and the McArdle disease iPSCs MA1 [28] and MA4 were differentiated towards innervated skeletal muscle cells following the protocol described by Mazaleyrat et al., 2020 [29]. With the progress of differentiation, some gradual changes in morphology in the cell culture were observed (Figure 1). At first, cells maintained some degree of stemness growing as colonies (Figure 1a), but soon cells with defined morphologies started to appear in the periphery (Figure 1b). Afterwards, the cells started aligning and fusing to form myofiber-like structures (Figure 1c–f), innervated thanks to the additional differentiation towards motor neurons. The innervated myogenic cells began to spontaneously contract around day 20, and they continued with stronger and more frequent contractions for all the processes of differentiation (Videos S1 and S2). This fact suggests the maturity and functionality of the differentiated cultures.

**Figure 2:**
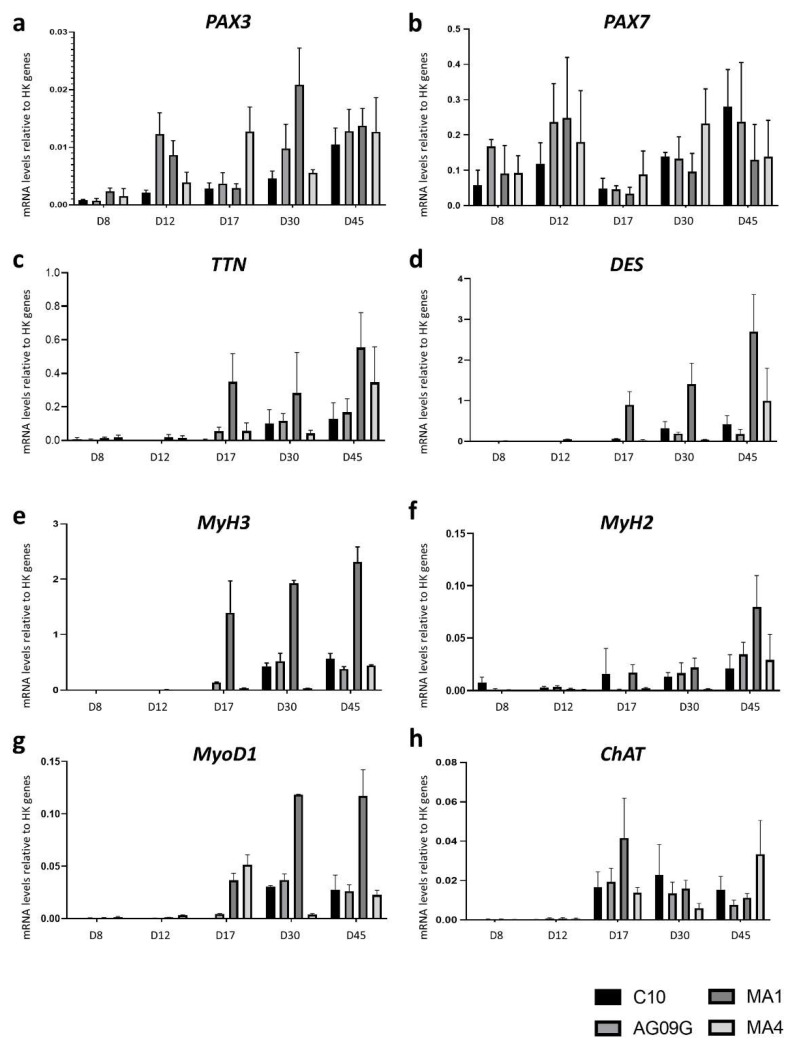
RT-qPCR analysis to assess the differentiation process from iPSCs to innervated skeletal muscle cells. The lines were evaluated at different time points during the differentiation process, at days (D) 8, 12, 17, 30 and 45. We analysed the evolution of the expression of different myogenic genes like *PAX3* (**a**)*, PAX7* (**b**)*, TTN* (**c**), *DES* (**d**), *MyH3* (**e**), *MyH2* (**f**) and *MyoD1* (**g**), in addition to the motor neuron marker *ChAT* (**h**). The values represent the mean of at least three replicates, and they are relative to the expression mean of two housekeeping (HK) genes (*HPRT* and *PPIA*). Error bars show standard deviation.

We evaluated the evolution of the expression of different myogenic and motor neuron markers along the differentiation process by RT-qPCR (Figure 2). The *PAX3* and *PAX7* genes code for transcription factors implicated in the cell induction of myogenic progenitors [30]. The *PAX7* gene is a key marker expressed in satellite cells, the stem cells present in skeletal muscle. We could observe how in our differentiated cultures the expression of these factors is maintained over the process, with *PAX7*^+^ cells present even at day 45 of differentiation (Figure 2a,b). This reflected the maintenance of a sufficient satellite cell population in the culture to assure its renewal. Titin and Desmin are related to the sarcomere structure, and they are markers of terminal differentiation [31]. We could observe the increase in their expression from day 17 onwards (Figure 2c,d). We also checked the expression of some isoforms of the myosin heavy chain, like the embryonic isoform (*MyH3*) and the adult isoform (*MyH2*), related to myotubes formation [32]. Both genes started to express around day 17 of differentiation, and although the embryonic isoform had a greater expression than the adult one, the detection of *MyH2* certified a mature differentiated culture (Figure 2e,f). The positive expression of *MyoD1*, a classic myogenic regulatory factor [33], was also confirmed (Figure 2g). Finally, we checked the expression of the motor neuron gene choline O-acetyltransferase (*ChAT*), which is related to the synthesis of acetylcholine [34]. Its expression was also maintained from day 17, when motor-neuron differentiation is induced with the Notch antagonist DAPT [29], onwards (Figure 2h).

**Figure 3:**
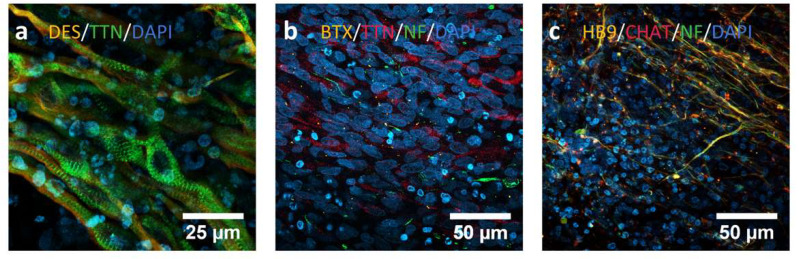
Immunocytochemistry analysis to assess the differentiation process from iPSCs towards innervated skeletal muscle. (**a**) C10 line at day 30, showing positive staining for Desmin (orange) and Titin (green), both mature myogenic markers. We can even observe a typical skeletal muscle alignment and striation pattern. (**b**) MA1 line at day 30. We can observe cells positive for Titin (TTN, red) as myogenic marker, and for neurofilament (NF, green) as motor neuron marker. The neuromuscular junctions are marked in orange with an α-bungarotoxin staining. (**c**) AG09G line at day 30. We can observe here the presence of motor neurons in the culture after the differentiation process, positive for markers like homeobox9 (HB9, orange), choline O-acetyltransferase (ChAT, red) and NF (green).

The expression of some key myogenic and motor neuron proteins was evaluated by immunocytochemistry at day 30 of differentiation (Figure 3). Differentiated cells expressed Titin and Desmin (sarcomere-related proteins) and they even exhibited a striated pattern and an aligned disposition typical of skeletal muscle cells (Figure 3a). The presence of neuromuscular junctions was also confirmed with an α-bungarotoxin (BTX) staining as we can observe in Figure 3b, with Titin-positive myogenic cells and Neurofilament-positive motor neuron cells. Finally, the presence of motor neurons in the culture was confirmed with the positive staining for markers like homeobox 9 (HB9), neurofilament (NF) and ChAT (Figure 3c).

**Figure 4:**
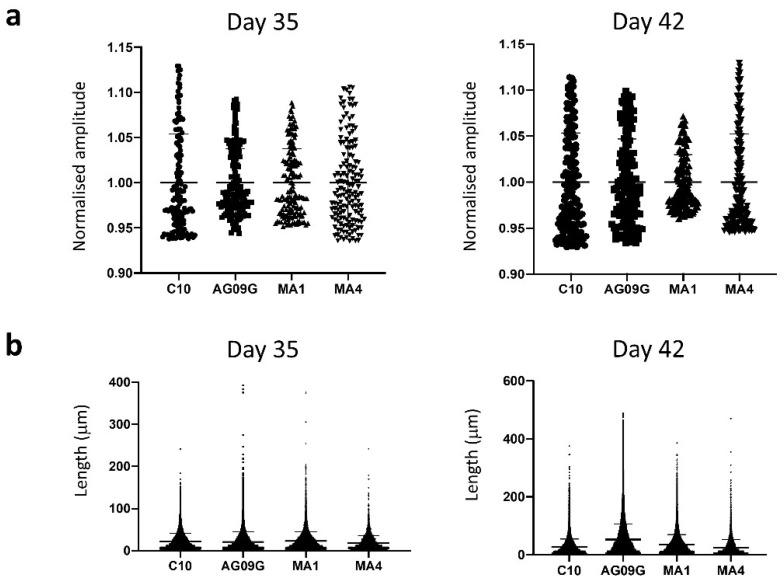
Functional assessment of the differentiated cultures. (**a**) Analysis of calcium transients. The characterization of calcium transients was performed at days 35 and 42 of differentiation, respectively, in the lines C10, AG09G, MA1 and MA4 by taking the values of intensity mean of at least 50 different fibres. There were no significant differences in the calcium transients of the four cell lines analysed (statistical analysis carried out with a Brown–Forsythe and Welch ANOVA test). (**b**) Contraction length analysis. We evaluated at least 4000 contraction events from three distinct differentiation experiments at days 35 and 42 in the lines C10, AG09G, MA1 and MA4. There were no significant differences between the values of contraction length of the four lines analysed (statistical analysis carried out with a Kruskal–Wallis test).

Subsequently, the functionality of the differentiated cultures was assessed using a fluorescent calcium-binding dye (FLUO-8^®^AM), which allowed to evaluate calcium transients and contraction capacity at days 35 and 42 (Videos S3 and S4). For calcium transients, we took the values of intensity mean of 50 different myogenic fibres from three distinct differentiation experiments. The normalized amplitude of the intensity mean values can be visualized in Figure 4a. There was no significant difference between the values of the distinct lines (statistical analysis performed using a Brown–Forsythe and Welch ANOVA test). In the case of contraction capacity, at least 4000 contraction events from three distinct differentiation experiments were evaluated. In Figure 4b, we can observe the representation of the contraction length values for all the lines evaluated. There were no significant differences between them (statistical analysis carried out with a Kruskal–Wallis test).

### 2.3. Validation of the McArdle Disease Model

**Figure 5:**
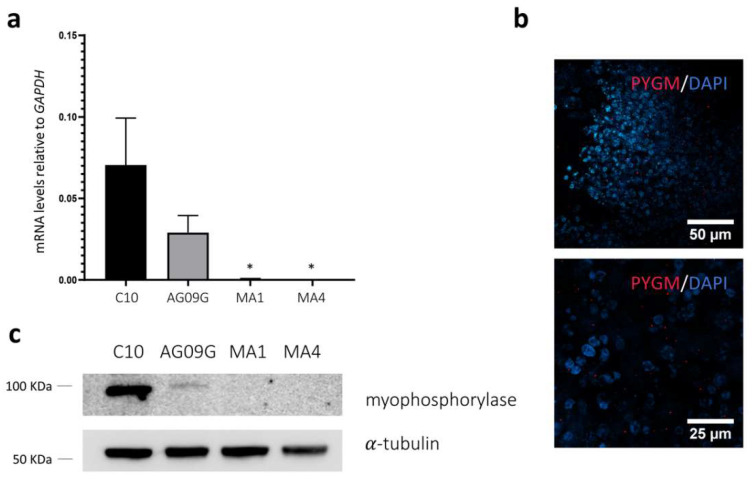
Evaluation of the expression of *PYGM* in the differentiated cell lines. (**a**) TaqMan™ assay to assess *PYGM* expression in the differentiated cell lines C10, AG09G, MA1, and MA4 at day 45. We can observe how the control cell lines do express *PYGM* as myogenic cultures, whereas both McArdle lines only maintain some negligible levels of expression. Values represent the expression mean of at least three independent replicates, relative to *GAPDH* as housekeeping gene. Error bars show standard deviation. * *p*-value < 0.05 versus control lines, one-way ANOVA with Holm–Sidak’s multiple comparisons test. (**b**) Immunocytochemistry analysis for the lines AG09G (above) and C10 (below). We can observe marked in red the presence of myophosphorylase in the differentiated cultures at day 30. (**c**) Western Blot analysis of the expression of myophosphorylase in the lines C10, AG09G, MA1 and MA4 at day 45 of differentiation. We can observe the presence of myophosphorylase in the control and its absence in the McArdle lines.

The validation of the created McArdle disease model was performed by evaluating the expression of *PYGM* and myophosphorylase after the differentiation protocol (Figure 5). First, we performed a TaqMan™ gene expression assay with *PYGM* as the target and *GAPDH* as the housekeeping gene at day 45 of differentiation in all the iPSC lines differentiated in this study. As we can observe in Figure 5a, both control lines (C10 and AG09G) did express *PYGM* after the differentiation, whereas the McArdle lines (MA1 and MA4) maintained only some negligible expression levels, with significant differences with respect to the control lines (statistical analysis carried out using a Holm–Sidak’s one-way ANOVA test with multiple comparisons). Additionally, an immunocytochemistry analysis revealed the expression of myophosphorylase at day 30 of differentiation for both the C10 and AG09G lines (Figure 5b). A Western Blot assay also confirmed the presence of myophosphorylase for C10 and AG09G and the absence for MA1 and MA4 (Figure 5c).

### 2.4. Gene Editing

#### 2.4.1. Design of the sgRNAs and Evaluation of their On-Target Efficiency

**Figure 6:**
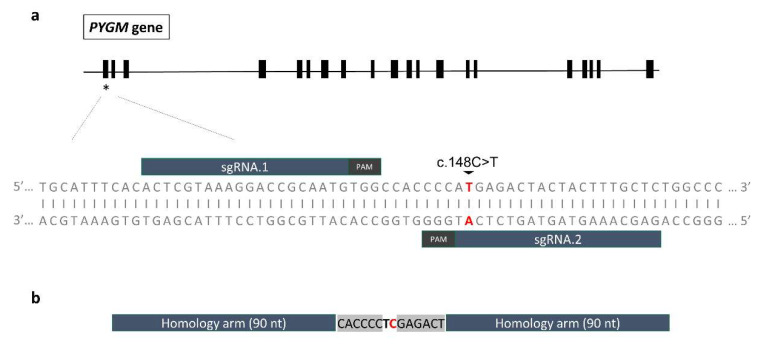
General scheme of the gene editing strategy. (**a**) Representation of the localization of the two designed sgRNAs targeting exon 1 of the *PYGM* gene. Both were selected to be placed as close to the c.148C>T; p.R50* mutation as possible, maintaining high on-target and low off-target activities. (**b**) ssODN repair template design. The 90-nt long homology arms leave two mutations in the centre: the edition of the c.148C>T; p.R50* target mutation (marked in red), and the introduction of a silent mutation to create a restriction site for the enzyme *Xho*I (c.147A>T; p.P49P, marked in bold).

To create the isogenic control for the previously generated iPS cell line IISHDOi001-A (named MA1 in this article) [20], two different sgRNAs to edit the *PYGM* c.148C>T; p.R50* mutation by CRISPR/Cas9 guided homology-directed repair (HDR) mechanisms were designed. For this purpose, two different design web pages (see the Materials and Methods section) were used to select the most suitable single guide RNAs (sgRNAs), based on their predictions of higher on-target and lower off-target activities. We considered only the guides that would induce a Cas9 cleavage close enough to the target mutation to enhance the editing event. The efficiency of two different sgRNAs (sgRNA.1 and sgRNA.2), both 20-nt long and with an NGG Protospacer Adjacent Motif (PAM) at the 3′ end of their sequence, was tested. Whereas sgRNA.1 was situated on the positive strand and would prompt a double-strand break 14 bp upstream of the mutation, sgRNA.2 was located on the negative strand and would induce the break just 1 bp downstream the mutation. The location of the two sgRNAs and their PAMs can be visualized in Figure 6a.

**Figure 7:**
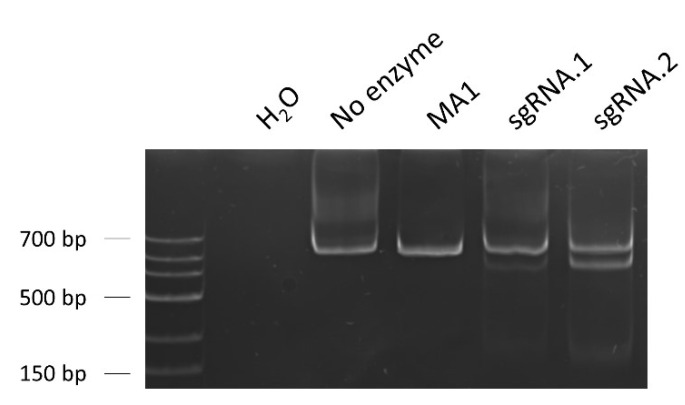
T7 endonuclease I assay to evaluate the on-target efficiency of the designed sgRNAs. The percentage of gene modification was calculated considering the band intensities of the T7-cleaved fractions, a reflexion of the Cas9 on-target activity. sgRNA.2 showed a greater on-target efficiency (28.8%) than sgRNA.1 (5.6%), being selected for the posterior edition experiments. Loading order: negative control (water as sample)—control without T7 enzyme—unedited iPSC line MA1—MA1 line nucleofected with Cas9/sgRNA.1 RNPs—MA1 line nucleofected with Cas9/sgRNA.2 RNPs.

To evaluate and compare the on-target efficiency of the designed sgRNAs, a classic T7E1 assay was performed. To that end, the iPSC line MA1 was nucleofected in the presence of ribonucleoproteins (RNPs) formed with the combination of a high-fidelity Cas9 and either sgRNA.1 or sgRNA.2. We extracted the genomic DNA for each case and amplified the region of interest with specific primers, obtaining a PCR product of 620 nt. After heteroduplex formation, the T7 endonuclease generates two cleaved fractions in the case of finding mismatches (reflection of the genome targeting, Cas9 cleavage, and posterior repair by different mechanisms). We estimated the frequency of gene modification by determining the percentage of T7-cleaved products. In our case, the calculated on-target efficacy for sgRNA.2 (28.8%) was higher than the one for sgRNA.1 (5.6%), (Figure 7). For that reason, sgRNA.2 was selected for the posterior edition experiments.

#### 2.4.2. Design of the ssODN Repair Template and Evaluation of the Edition Efficiency

In the case of single-nucleotide editions via HDR mechanisms, it has been demonstrated that single-stranded oligodeoxynucleotides (ssODNs) give rise to better efficiency [21]. Considering this, and having selected the best sgRNA, we designed a ssODN repair template to correct the *PYGM* c.148C>T; p.R50* mutation (Figure 6b). The 90-nt long homology arms of the ssODN employed in this study leave in the core two-point mutations: the first one would be the correction of the *PYGM* c.148C>T; p.R50* mutation, whereas the second one would be the introduction of a silent mutation (c.147A>T; p.P49P) in the sequence. This second modification would prevent the Cas9 from further cleavage as it would introduce a mismatch in the sequence. Additionally, it would create a restriction site for the enzyme *XhoI* to be used for the estimation of the edition efficiency by Restriction Fragment Length Polymorphism (RFLP). That way, if the edit had taken place correctly, the enzyme *XhoI* would digest the PCR amplicon and would generate two bands of 452 and 168 nt long.

**Figure 8:**
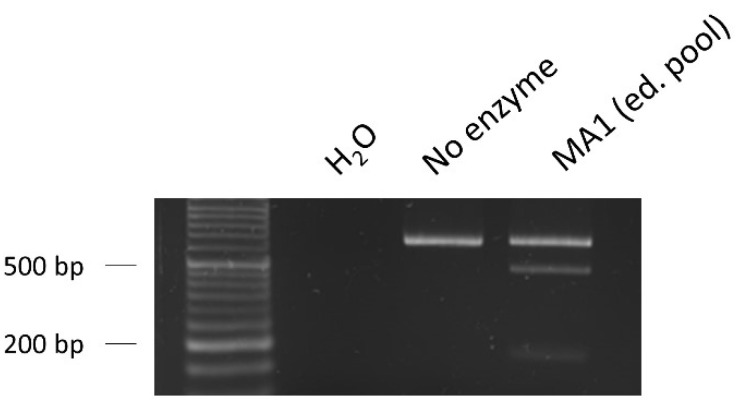
RFLP assay to evaluate the editing efficiency. The percentage of edit (30%) was calculated based on the band intensities of the fraction cleaved by the restriction enzyme *XhoI*, showing an acceptable number of edited cells present in the pool. Loading order: negative control (water as sample)—control without *XhoI* enzyme—edited pool of the iPSC line MA1 nucleofected with Cas9/sgRNA.2 RNPs and ssODN repair template.

For the gene editing, iPSCs were nucleofected in the presence of Cas9/sgRNA.2 RNPs and the ssODN repair template to force HDR mechanisms to edit the sequence as desired. After the expansion of the edited pool, we extracted genomic DNA, amplified the region of interest, and performed a digestion with the enzyme *XhoI*. As we can observe in Figure 8, RFLP showed a high editing efficiency (30%), revealing that the edit had taken place correctly in a relatively high percentage of cells.

#### 2.4.3. Subcloning and Verification of the Gene Edition

**Figure 9:**
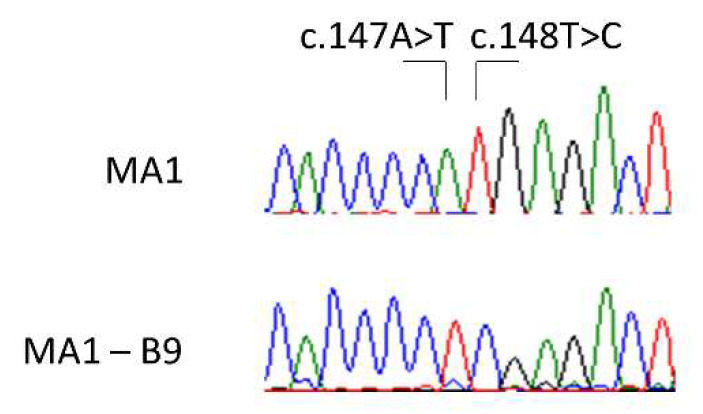
Validation of the successful edition of the selected clone MA1—B9 (below) by Sanger sequencing, compared to the MA1 origin line (above). We can observe both the modifications introduced in the edited sequence: c.147A>T and c.148T>C.

After the confirmation of the gene editing by RFLP, subcloning of the nucleofected iPSCs pool was carried out. At this point, it was particularly important to assure that the clones generated came from a single cell, otherwise the established lines could be a mixing of successfully edited cells and either unedited or wrongly edited cells. We evaluated the edit event in 96 different clones by Sanger sequencing, finding evidence of a correct edit in 26 of them. We selected the edited clone B9 for further experiments, as it showed a typical embryonic stem cell-like morphology and normal growth behaviour. After expanding the clone, the edit was checked again by Sanger sequencing (Figure 9). The established edited line was named MA1-B9.

#### 2.4.4. Off-Targets Analysis

**Figure 10:**
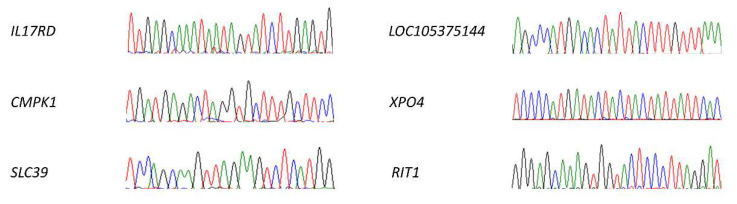
Off-targets analysis. We selected six different off-targets (*IL17RD*, *CMPK1*, *SLC39*, *LOC105375144*, *XPO4*, and *RIT1*), being the most likely ones to be modified according to the prediction algorithms. We evaluated the possible variations in their sequence by Sanger, finding no modifications in any of them. The areas shown in the image correspond to the sequences analogous to sgRNA.2, all of them identical to the reference sequence.

One of the main concerns with the CRISPR technology is the possibility of modifying the sequence of undesired targets. We tried to minimise this with different strategies, like delivering the CRISPR system using RNPs, selecting a high-fidelity Cas9, or designing carefully the sgRNAs. However, the chances of off-target modifications are always present. We selected six off-targets between the most probable ones predicted by the two design pages employed in this study (see Materials and Methods section). All of them were located in either coding sequences or RNA genes and had between three and four mismatches (the selected guides did not have possible off-targets with a lower number of mismatches). We amplified each region of interest and we sequenced all of them by Sanger. None of the selected off-targets presented any modification (Figure 10).

#### 2.4.5. Pluripotency and Integrity Assessment

To evaluate if the edited iPSC line MA1-B9 preserved its integrity and stem cell features, a battery of assays was performed. First, the positive expression of several stemness markers (OCT4, NANOG, and TRA-1-81) was confirmed by immunofluorescence (Figure S3a). Second, RT-qPCR analysis of *OCT4*, *SOX2*, *CRIPTO*, *NANOG*, and *REX1* also verified the pluripotency of the line. To this end, total RNA from human embryonic stem cells (hESCs, Celprogen) and from the iPSC line IISHDOi007-A [22] were used as a reference for the gene expression levels of stemness markers (Figure S3b).

In addition, an in vitro differentiation assay was carried out as functional evidence of pluripotency. As shown in Figure S3c, the edited line can generate cell types related to the three germ layers: mesoderm (positive for SMA), endoderm (AFP), and ectoderm (positive for Tuj1). The MA1-B9 also presented a normal karyotype 46, XX (Figure S3d), revealing that the edition protocol had not induced the appearance of karyotypic abnormalities. The line was also checked to be mycoplasma-free (Figure S3e). Finally, a DNA fingerprinting was performed to prove the genetic identity of the isogenic control MA1-B9 from the previously created iPSC line MA1 (IISHDOi001-A; data available upon request).

### 2.5. Pilot Study to Evaluate Two Read-Through Compounds in the iPSC-Based McArdle Disease Model

**Figure 11:**
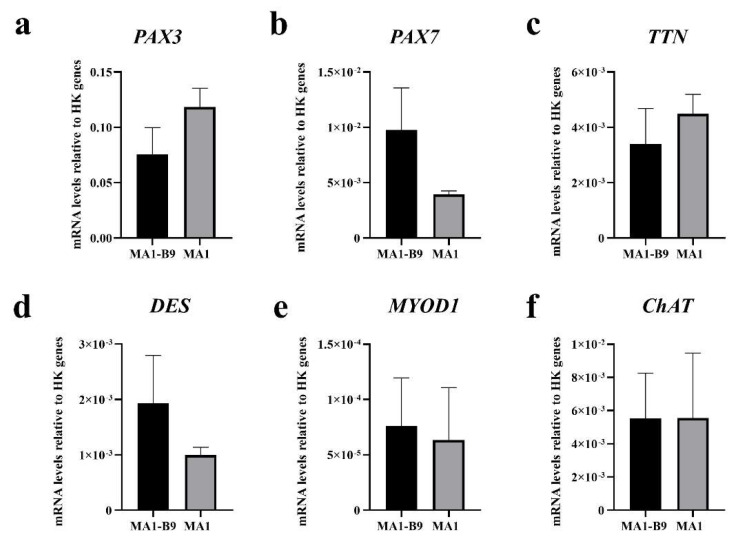
Analysis by RT-qPCR of the expression of myogenic and motor neuron genes to evaluate the process of differentiation at day 40 of the lines MA1-B9 and MA1. The expression of the genes *PAX3* (**a**), *PAX7* (**b**)*, TTN* (**c**)*, DES* (**d**), and *MYOD1* (**e**) as myogenic markers, along with *ChAT* (**f**) as a motor neuron marker, was assessed. The positive expression of all of them certifies the presence of myogenic cells and motor neurons in the differentiated culture. The values represent the mean of at least three replicates, and they are relative to the expression of two housekeeping (HK) genes: *HPRT* and *PPIA*. Error bars show standard deviation.

The isogenic iPSC model for McArdle disease (composed by the line MA1-B9 as isogenic control and the line MA1 as McArdle line harbouring the mutation c.148C>T; p.R50* in the *PYGM* gene) was employed to evaluate the action of two read-through compounds as possible therapy. First, both lines were differentiated towards innervated skeletal muscle using the protocol developed by Mazaleyrat et al. [29]. After 40 days of differentiation the expression of myogenic markers (*PAX3*, *PAX7*, *TTN*, *DES* and *MYOD1*) and a motor neuron marker (*ChAT*) was evaluated by RT-qPCR in both lines. A positive expression of all the markers assessed was observed, certifying that the process of differentiation had originated both myogenic and motor neuron cells in the culture (Figure 11).

Subsequently, both differentiated lines were treated between days 41 and 43 of differentiation with two read-through drugs: PTC124 (treatment at 2, 5, 10, and 50 μM, in absence or presence of 0.5 mM caffeine) and RTC13 (treatment at 2, 5, 10, and 50 μM).

**Figure 12:**
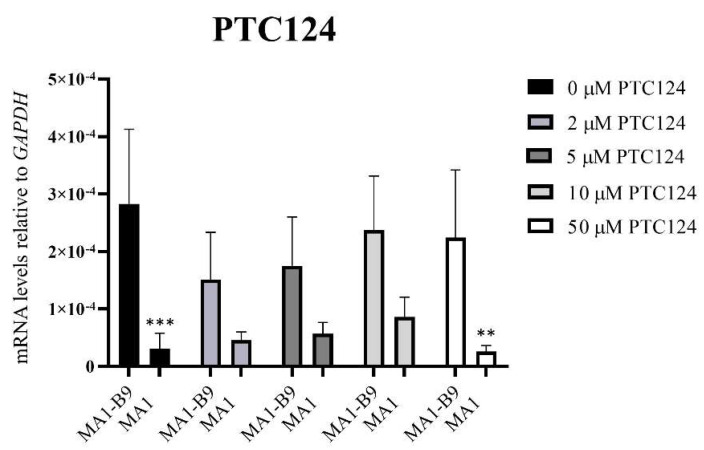
*PYGM* expression analysis using TaqMan™ assays after the treatment with different concentrations of PTC124 (2, 5, 10, and 50 μM) in the differentiated lines MA1-B9 and MA1. We observed no significant differences with the treatments at 2, 5, and 10 μM between the control and the mutant line. The values are representative of at least three independent replicates, relative to *GAPDH* as housekeeping gene. Error bars show standard deviation. The statistical analysis was performed using a two-way ANOVA analysis with Sidak’s multiple comparisons test between the control and the mutant line for each of the concentrations (** *p*-value < 0.01 and *** *p*-value < 0.001). None of the evaluated concentrations showed significant differences with respect to the absence of treatment in the control line (*p*-value > 0.05, two-way ANOVA analysis with Sidak’s multiple comparisons test).

**Figure 13:**
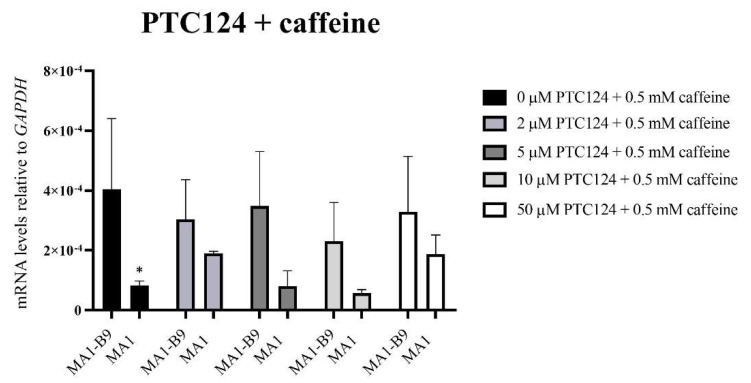
*PYGM* expression analysis using TaqMan™ assays after the treatment with different concentrations of PTC124 (2, 5, 10, and 50 μM) in conjunction with 0.5 mM caffeine in the differentiated lines MA1-B9 and MA1. We observed no significant differences with all the treatments evaluated between the control and the mutant line. The values are representative of at least three independent replicates, relative to *GAPDH* as housekeeping gene. Error bars show standard deviation. The statistical analysis was performed using a two-way ANOVA analysis with Sidak’s multiple comparisons test between the control and the mutant line for each of the concentrations (* *p*-value < 0.05). There were no differences between each concentration and the absence of treatment for the line MA1-B9 (*p*-value > 0.05, two-way ANOVA analysis with Dunnet’s multiple comparisons test).

**Figure 14:**
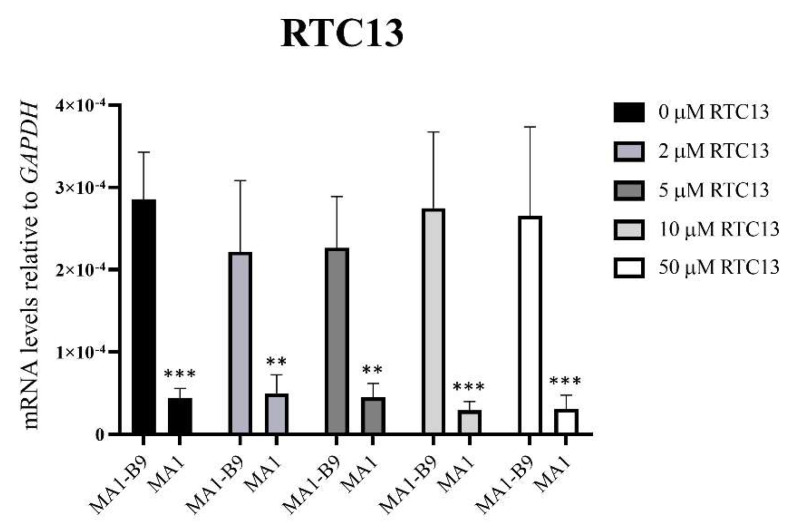
*PYGM* expression analysis using TaqMan™ assays after the treatment with different concentrations of RTC13 (2, 5, 10, and 50 μM) in the differentiated lines MA1-B9 and MA1. None of the assessed concentrations rescued the levels of *PYGM* expression in the mutant line in comparison to the isogenic control. The values are representative of at least three independent replicates, relative to *GAPDH* as housekeeping gene. Error bars show standard deviation. The statistical analysis was performed using a two-way ANOVA analysis with Sidak’s multiple comparisons test between the control and the mutant line for each of the concentrations (** *p*-value < 0.01 and *** *p*-value < 0.001). None of the evaluated concentrations showed significant differences with respect to the absence of treatment in the control line (*p*-value > 0.05, two-way ANOVA analysis with Dunnet’s multiple comparisons test).

Cell pellets were collected after 24 h of treatment. A TaqMan™ assay was used to evaluate the possible rescue of *PYGM* expression after the treatments with the different drugs: PTC124 (Figure 12), PTC124 with caffeine (Figure 13), and RTC13 (Figure 14). The vehicle employed for the dilution of the drugs (DMSO) did not induce any type of effect or alteration in the differentiated lines. Statistical studies were performed with a Sidak or Dunnet’s multiple comparisons tests, comparing the expression levels between the mutant and the control lines. We also assessed that there were no significant differences with respect to the absence of treatment (0 μM). In the case of PTC124 (Figure 12), with 2, 5, and 10 μM treatment, there were no significant differences between the lines MA1-B9 and MA1, with a possible tendency to increase *PYGM* expression in the mutant line. The combined treatment with PTC124 and caffeine (Figure 13) rescues *PYGM* levels in the MA1 line with all the concentrations assessed. Regarding RTC13 (Figure 14), none of the tested concentrations equated to the expression levels of the lines MA1-B9 and MA1. There were no significant differences between each treatment and its absence for all the drugs in the line MA1-B9.

## 3. Discussion

McArdle disease is an autosomal recessive rare disorder in which glycogen breakdown is blocked in skeletal muscle, impairing the use of glycogen as the primary energy source during exercise and causing a major exercise intolerance [1]. The lack of an ideal human in vitro model for the disease could be one of the main reasons why there is no treatment associated. The iPSC-based models are a very promising alternative to recapitulate in a dish the main features of a disease, as they are generated from somatic cells of a patient, they have auto-renewal capacity and they can be differentiated towards the specific tissue or tissues affected [35]. Skeletal muscle is one of the most complex tissues in the human body, with an extraordinary degree of organization and molecular intricacy [36]. Perhaps due to this complexity, skeletal muscle has always represented a difficult tissue to be modelled in vitro. Additionally, its embryonic development and maturation are still not fully understood [37]. Up to now, several protocols have been published to differentiate iPSCs to skeletal muscle tissue following distinct strategies [38]; however, the selection of the best protocol should be performed carefully, considering its safety and efficacy, among other parameters.

The main aim of this study was the establishment of an iPSC-derived skeletal muscle model of McArdle disease, which could be a valuable tool for the search of a therapy against this disease. For this purpose, two control and two McArdle iPSC lines were differentiated to innervated skeletal muscle cells. A protocol that induces a double differentiation towards skeletal muscle and motor neurons was selected [29], based on the supplementation of small molecules and defined factors to the culture media. The success of the differentiation strategy was confirmed with positive expression of typical myogenic and motor neuron markers, assessed by RT-qPCR and immunofluorescence. The presence of calcium transients and contraction capacity also suggested the functionality and maturity of the generated skeletal muscle cultures.

Myogenic and motor neuron gene expression levels differ between the cell lines employed in this study. This can be explained by the type of differentiation protocol selected. With the addition of selected transcription factors and small molecules in the culture media, we can only direct the differentiation towards a certain lineage, but each line can respond differently to these stimuli. This only emphasizes the need to standardise the differentiation protocols and develop techniques without line-to-line fluctuations.

Contraction force is a hallmark trait previously reported to be reduced in McArdle disease patients [39] and in the McArdle mouse model [40]. Additionally, the sarcoplasmic reticulum calcium ATPase 1 (SERCA1) has been described to be downregulated in McArdle patients, which could prejudice calcium transport in type II muscle fibres [41]. However, when we analysed the possible differences between calcium transients and contraction capacity between the controls and the McArdle lines, we found no significant differences. This could be attributed to the differences between the disorder itself and the aspects that indeed can be modelled in vitro. Perhaps forcing the model to only use glycogen as energetic source (removing glucose from the medium, for instance) could constitute a way to bring the model closer to the disease and detect possible differences between McArdle and control lines at this level.

The lack of myophosphorylase protein in muscle is another distinctive feature of patients with McArdle disease carrying the p.R50* mutation [42]. We confirmed the absence of myophosphorylase in the McArdle iPSC-derived skeletal muscle model and the presence in the control ones after the differentiation process, validating the generated model. Expression of *PYGM* would not be expected due to nonsense-mediated decay (NMD) mechanisms, previously confirmed to occur in cells harbouring the nonsense mutation c.148C>T; p.R50* [43]. We have observed that whereas the expression of *PYGM* was clear in the control lines, we still detected some residual expression in the McArdle lines. As we were employing TaqMan™ probes, perhaps their sensitivity still allows us to detect residual transcripts not yet removed, or maybe NMD mechanisms are not fully understood in these cells. There is also a difference in the levels of *PYGM* expression between both control lines, C10 and AG09G, with consistent results confirmed by RT-qPCR and Western Blot. This could be due to the different genetic background of the lines, or even to the differentiation process itself, which can differ between the lines as remarked above. Anyhow, the presence of myophosphorylase in the controls and absence in the McArdle lines, proved by immunodetection, validates the iPSC-based model of McArdle disease. This way, the possible rescue of myophosphorylase expression in this model could be used as a readout to evaluate potential drug candidates.

Nowadays, the use of isogenic control iPSCs has become an ideal tool to be used for drug screenings, as they eliminate the genetic background as a modifier. CRISPR/Cas9 technology has opened the door to not only editing but also regulating and targeting the genome in an easier, faster and more elegant fashion [44]. Using this technology, the establishment of isogenic controls can be performed very easily. In this study, we report the generation of an isogenic control of the iPSC line MA1 using CRISPR/Cas9 technology. For this purpose, we targeted exon 1 of the *PYGM* gene using RNPs formed by the enzyme Cas9 and a pre-designed sgRNA with good on-target efficiency, in the presence of a ssODN repair template to drive HDR mechanisms to edit the genome in the desired way. The edited line (MA1-B9) maintained its pluripotency and integrity after the editing protocol. Additionally, six different possible off-targets were evaluated by Sanger sequencing, finding no modifications in any of them.

Using the isogenic skeletal muscle McArdle iPSC-based model, we performed a pilot study to evaluate the possible rescue of myophosphorylase expression after the treatment with two read-through compounds: PTC124 (with and without caffeine, as caffeine is supposed to enhance its effect [45]) and RTC13. The treatment with 2, 5, and 10 μM of PTC124 increased the *PYGM* expression levels in the McArdle line, like with all the conditions tested in the presence of caffeine. However, none of the concentrations of RTC13 rescued the levels of expression in the McArdle lines. The results obtained with PTC124 contrast with a previously published study [46]. In this study, the authors evaluated the efficiency of different read-through compounds (including PTC124) in three different cellular models. These models were primary skeletal muscle cells derived from the McArdle murine model, HeLa cells transiently transfected with p.R50* plasmid constructs or HEK293T cells stably expressing these constructs. The differences observed in terms of PTC124 read-through efficiency could be explained as these models may diverge from the actual human disorder due to inter-species differences or because of constituting cell lineages distinct from the one mainly affected in McArdle disease, which are skeletal muscle cells. Thus, the skeletal muscle iPSC-based model, reported in this work, may be closer to reality in this sense, offering a more accurate approach to the human disorder. Although our study is still preliminary, a deeper analysis of the effects of PTC124 with and without caffeine (or even other read-through compounds) should be performed in the generated model, as they could be a promising therapy.

## 4. Materials and Methods

This study was reviewed and approved by the Institutional Ethical Committee of the “Instituto de Investigaciones Biomédicas Alberto Sols”, CSIC-UAM, 406 329 1.

### 4.1. Establishment of the iPSC Lines C10 and MA4: Cell Reprogramming

We reprogrammed primary fibroblasts from a commercial source (Lonza, CC2509) to generate the control iPSC line named C10, and fibroblasts from a McArdle patient harbouring the *PYGM* mutation c.148C>T; p.R50* in homozygosis for the iPSC line MA4. We used the CytoTune-iPS 2.0 Sendai reprogramming kit (Thermo Fisher Scientific, Waltham, MA, USA; #A16517), following the manufacturer’s instructions. After the expansion of the reprogrammed lines, we selected two of them to continue the battery of pluripotency and integrity tests. The control line C10 has been originated from the same source as the previously published line IISHDOi002-A [27], whereas the line MA4 comes from the same fibroblasts as the published line IISHDOi001-A (named in this article as MA1) [28]. The maintenance and expansion of the iPSC lines were performed both on feeder and feeder-free conditions, as described by Galera et al., 2016 [47]. The presence of the *PYGM* mutation c.148C>T; p.R50* in the reprogrammed McArdle line was confirmed by Sanger sequencing.

### 4.2. Pluripotency and Integrity Assessment of the iPSC Lines

#### 4.2.1. Alkaline Phosphatase Staining

The positive alkaline phosphatase staining was assessed using the phosphatase alkaline blue membrane substrate solution kit (Merck, Darmstadt, Germany; #AB0300), following the instructions provided by the manufacturer.

#### 4.2.2. Immunocytochemistry for Pluripotency Assessment

Cells were fixed with 4% paraformaldehyde for 30 min at room temperature (RT) and permeabilized with 0.1% Triton X-100 in Tris-buffered saline (TBS) for 45 min at RT. Blocking was carried out with 3% donkey serum, 0.3% Triton in TBS for 2 h at RT. Primary antibodies were incubated overnight at 4 °C and secondary antibodies at RT for 2 h in the dark (Table 1). Nuclei were counterstained with DAPI (Merck, Darmstadt, Germany; #28718-90-3).

#### 4.2.3. RT-qPCR for Pluripotency Assessment

We performed the RNA extraction with TRI^®^ Reagent Solution (Invitrogen, Waltham, MA, USA; #AM9738). The retrotranscription of 1 μg of RNA was carried out using the RevertAid RT Reverse Transcription Kit (Thermo Fisher, Waltham, MA, USA; #K1691). The qPCR amplification was performed in a 7500 Fast Real-Time PCR System, by Applied Biosystems (Waltham, MA, USA), using the GoTaq^®^ qPCR Master Mix (Promega, Madison, WI, USA; #A6002) following the instructions provided by the manufacturer. The expression levels were normalized relative to *GAPDH*, and they represent at least three independent replicates. The sequence of the qPCR primers employed can be found in Ortuño–Costela et al., 2017 [28].

#### 4.2.4. In Vitro Differentiation

We followed the protocol described by Galera-Monge et al., 2019 [48] for the embryoid bodies generation and posterior in vitro spontaneous differentiation to cell types of the three germ layers. The positive expression of selected markers was assessed by immunocytochemistry as described in Section 4.2.3, using the antibodies listed in Table 2: α-fetoprotein (AFP) for endoderm, α-smooth muscle actin (SMA) for mesoderm, and β-III-tubulin (Tuj1) for ectoderm.

#### 4.2.5. Elimination of the Sendai Virus

The removal of the Sendai virus employed to deliver the pluripotency factors inside the cells was assessed by RT-PCR. For this purpose, total RNA extraction and retrotranscription were performed as described in Section 4.2.3, followed by a PCR reaction designed according to the specific recommendations provided by the CytoTune-iPS 2.0 Sendai reprogramming kit (Invitrogen, Waltham, MA, USA; #A16517).

#### 4.2.6. Karyotype Analysis

For the evaluation of the karyotype, cells with more than 20 passages were treated with 10 μg/mL of Colcemid™ (Gibco, Waltham, MA, USA; #15212012) for 90 min at 37 °C and trypsinised. Cells were then placed in a KCl 0.075 M hypotonic solution and fixed with Carnoy’s solution, being dropped on a microscope glass slide. Wright staining was used for the G-banding, analysing at least 20 metaphases. The FISH analysis was performed with specific DNA probes for centromeric regions DXZ1/DYZ1. Slides were denatured at 72 °C for 2 min and incubated at 37 °C for 16 h for hybridization. Afterwards, they were washed in SSC with 0.1% Tween-20 and mounted using DAPI. A Nikon fluorescent microscope was used to analyse 200 interphase cells. Digital images were acquired with a monochrome CCD camera linked to Metasystem software.

#### 4.2.7. Mycoplasma Detection

The established iPSC lines were evaluated to be mycoplasma-free by PCR. To this aim, the supernatant of a 3-day confluent culture was boiled at 95 °C for 5 min and centrifuged at 13,000 g for 5 sec. For the PCR we used the primers MGSO-Fw (5′-TGCACCATCTGTCACTCTGTTAACCTC-3′) and GPO-Rv (5′-GGGAGCAAACAGGATTAGATACCCT-3′). The product of the PCR was resolved in a 1% agarose gel. The band at 300 bp represents that the sample is positive for mycoplasma.

#### 4.2.8. STR Analysis

A DNA fingerprinting analysis was performed to demonstrate the origin of the established iPSC lines from the starting fibroblasts. The subsequent markers were amplified by PCR, followed by a fragment analysis: D13S317, D7S820, VWA, D8S1179, D21S11, D19S433, D2S1338, and amelogenin for sex determination. The analysis was carried out using the software ABI PRISM 3100 Genetic analyser and Peak Scanner v3.5 (Applied Biosystems, Waltham, MA, USA). The PCR primers can be consulted in Ortuño–Costela, et al., 2017 [28].

### 4.3. iPSC Culture

The iPSCs were cultured at 37 °C and 5% CO_2_ on hESC-qualified Matrigel-coated plates (Corning, New York; #354277) using mTeSR™1 medium (StemCell Technologies, Vancouver, Canada; #85850). The cells were dissociated and passaged when they reached about 80% confluence using RELeSR™ (StemCell Technologies, Vancouver, Canada; #100-0484), following the instructions of the manufacturer.

### 4.4. Differentiation to Innervated Skeletal Muscle Cells

The differentiation to innervated skeletal muscle was performed following the protocol previously described by Mazaleyrat et al., 2020 [29]. After reaching a high confluence, iPSCs growing in mTeSR™1 were manually picked and seeded on hESC-qualified Matrigel-coated plates. For the differentiation of iPSCs to skeletal muscle cells the basal medium for the maintenance of the cells is composed by Neurobasal™ (Gibco, Waltham, MA, USA; 21103-049), supplemented with GlutaMAX™ 1× (Invitrogen, Waltham, MA, USA; 35050-038), *Penicillin-Streptomycin* 1× (Gibco, Waltham, MA, USA; 15140122), non-essential amino acids 1× (Merck, Darmstadt, Germany; M7145), N-2 supplement 1× (Gibco, Waltham, MA, USA; 17502048) and B-27™ supplement 1× (Gibco, Waltham, MA, USA; 17504044). To induce myogenic and motor neuron differentiation subsequent medium daily changes were performed with the basal medium supplemented with the compounds shown in Table 3 [49]. The iPSC lines which were differentiated towards skeletal muscle cells in this study were C10 [27] and AG09G (kindly gifted by Dr Magdinier’s group) as control lines and MA1 [28] and MA4 as McArdle lines.

#### 4.4.1. Immunocytochemistry for Differentiation Assessment

Cells for immunocytochemistry were fixed at day 30 of differentiation with 4% paraformaldehyde overnight at 4 °C. Permeabilization and blocking were performed with 3 % BSA, 0.4 % Triton in phosphate-buffered saline (PBS) for 1 h at RT. Incubation with primary antibodies was performed overnight at 4 °C, and with the secondary antibodies at RT for 1 h in the dark, both diluted in blocking solution (Table 4). The mounting of the slides was performed using Vectashield^®^ mounting medium with DAPI to stain the nuclei (Vector laboratories, Newark, CA; #H-1200-10). For detection of neuromuscular junctions, we performed a staining using Alexa Fluor^®^ 555 α-bungarotoxin conjugate diluted 1:400 (Thermo Fisher Scientific, Waltham, MA, USA; #B35451).

#### 4.4.2. RT-qPCR for Differentiation Assessment

Cell pellets were obtained at different time points during the differentiation process (days 8, 12, 17, 30, and 45, respectively) using RELeSR™ (StemCell Technologies, Vancouver, Canada; #100-0484). Total RNA extraction was performed using the RNAeasy kit (Qiagen, Hilden, Germany; #74104) following the manufacturer’s instructions. We carried out the retrotranscription of 1 μg of RNA using the Superscript IV kit (Invitrogen, Waltham, MA, USA; #18091200) according to the instructions provided by the manufacturer. The qPCR was performed using LightCycler^®^ 480 SYBR Green I Master (Roche, Basel, Switzerland; #04707516001) with 1 μL of the retrotranscription product, following the manufacturer’s instructions. All the values are representative of at least three independent replicates, and the expression levels were normalized to the mean of two different housekeeping genes (*HPRT* and *PPIA*). The qPCR primers are listed in Table 5.

#### 4.4.3. Calcium Transients and Contraction Capacity Analyses

Calcium transients and contraction capacity were analysed at days 35 and 42 of differentiation. Cells were incubated at 37 °C for 30 min in the presence of 5 μM FLUO-8^®^AM fluorescent Ca^2+^ binding dye (AAT Bioquest, #1345980-40-6) and 0.08 % Pluronic acid. After exciting at 488 nm, fluorescence images were taken as time series at different regions in an Imaging Observer system (Axio Observer.Z1/7, Zeiss, Jena, Germany) at 10x (151 images in total for 30 sec with 200 msec intervals). The analysis was performed using the Imaris 9.9 software (Oxford Instruments, Abingdon, UK). Calcium transients were analysed taking the values for intensity mean of 50 different fibres representative of at least three separate differentiation experiments. We performed a multiple comparison using a Brown–Forsythe and Welch ANOVA test to search for statistical differences. For contraction analysis, we took the values of contraction length of a minimum of 4000 different contraction events from at least three separate differentiation experiments. The statistical analysis was performed using a Kruskal–Wallis test.

### 4.5. Validation of the Skeletal Muscle iPSC-Based Model for the Study of McArdle Disease

#### 4.5.1. RT-qPCR

For the expression analysis of *PYGM*, we designed a TaqMan^®^ gene expression assay. We performed a duplex assay with two different probes, in conjunction with the TaqMan^®^ gene expression master mix (Applied Biosystems, Waltham, MA, USA; #4369016): *PYGM*-FAM as target (assay ID Hs00989942_m1, #10794597) and *GAPDH*-VIC as housekeeping gene for the normalization of the expression levels (assay ID Hs02786624_g1, #11957021), both from Applied Biosystems (Waltham, MA, USA). The qPCR with the TaqMan^®^ probes was performed in a 7500 Fast Real-Time PCR system (Applied Biosystems, Waltham, MA, USA). The values represent the mean of at least three replicates. The statistical study was performed with a one-way ANOVA Holm–Sidak’s multiple comparisons test, considering as statistically significant a *p*-value < 0.05.

#### 4.5.2. Western Blot

We evaluated the expression of myophosphorylase using a Western Blot analysis. Cell pellets were collected at day 45 of differentiation using RELeSR™ (StemCell Technologies, Vancouver, Canada; #100-0484). Protein extraction was carried out using a lysis buffer (50 mM Tris-HCl pH 7.5, 150 mM NaCl, 5 mM EDTA, 0.1 % SDS) with protease inhibitors (Roche, Basel, Switzerland; #11873580001). Protein quantification was performed using the DC™ protein assay kit (Bio-Rad, Hercules, CA, USA; #5000112), according to the manufacturer’s instructions. We performed an SDS-PAGE in 4–20% Mini-PROTEAN^®^ TGX™ precast protein gels (Bio-Rad, Hercules, CA, USA; #4561094) and transferred the proteins to a nitrocellulose membrane (Bio-Rad, Hercules, CA, USA; #1704158) with the Trans-Blot Turbo Transfer system by Bio-Rad. The blocking of the membrane was performed with 5% non-fat dry milk, 0.1 % Tween-20 (Merck, Darmstadt, Germany; #P1379) in TBS for 1 h at RT. The PYGM primary antibody (Merck, Darmstadt, Germany; #HPA056003) was diluted 1:50 in 0.1 % Tween-20, 1 % non-fat dry milk in TBS, with an incubation period of 17 h at 4 °C with gentle agitation. GARPO secondary antibody (Molecular Probes, Eugene, OR, USA; #G21234) was diluted 1:2500 in the same solution as the primary antibody, and it was incubated at RT for 1 h. The membrane was revealed with Clarity Max™ Western ECL Blotting Substrates (Bio-Rad, Hercules, CA, USA; #1705062). α-Tubulin-HRP (Abcam, Cambridge, UK; #ab40742) was used as a loading control, diluted 1:5000. Western Blot analyses were performed by Dr García-Consuegra, from the Proteomics Service in the Research Institute of Hospital 12 de Octubre. 

#### 4.5.3. Immunocytochemistry

The assessment of the myophosphorylase expression by immunocytochemistry was performed following the protocol described in Section 4.4.1, using the primary antibody rabbit anti-PYGM (Merck, Darmstadt, Germany; #HPA056003) at a 1:100 dilution.

### 4.6. Gene Editing with CRISPR/Cas9

#### 4.6.1. Design of the sgRNAs

We designed the crRNA domain of two possible sgRNAs (named sgRNA.1 and sgRNA.2), targeting exon 1 of the *PYGM* gene. For this purpose, we took into account the proximity of the guides to the c.148C>T; p.R50* mutation, along with the predictions of higher on-target efficiency and lower possibility of off-targets modifications provided by two different tools: the design page integrated by IDT “https://eu.idtdna.com/site/order/designtool/index/CRISPR_CUSTOM (accessed on 30 January 2022)” and the prediction software CRISPOR [50]. The sequence of the selected crRNA domains is specified in Table 6, along with the 3′-situated PAM for each case.

The tracrRNA domain was acquired separately (IDT, Newark, NJ, USA; #1072532). The crRNA and the tracrRNA domains were assembled into the complete sgRNA by mixing both at equimolar concentrations and annealing at 95 °C for 2 min.

#### 4.6.2. Design of the ssODN

A ssODN repair template for the correction of the *PYGM* c.148C>T; p.R50* mutation via the HDR pathway was designed. An additional silent mutation (c.147A>T; p.P49P) was introduced as well to prevent Cas9 from further cleavage and to create a restriction site for the enzyme *XhoI*, facilitating the posterior evaluation of the edition efficiency. This other change did not introduce any additional splicing sites, assessed with the NetGene2 server “http://www.cbs.dtu.dk/services/NetGene2 (accessed on 15 February 2022)” [51]. The ssODN, with 90 nt-long homology arms at both sides of the modifications, was ordered as an Ultramer oligonucleotide to IDT. The complete sequence of the ssODN is specified below (the correction of the c.148C>T mutation is marked in bold, and the new silent mutation introduced is underlined, flanked by the homology arms).

Homology arm 1:

GCCGGCGTGGAGAACGTGACTGAGCTGAAAAAGAACTTCAACCGGCACCTGCATTTCACACTCGTAAAGGACCGCAATGTGGCCACCCC

Core: T**C**

Homology arm 2:

GAGACTACTACTTTGCTCTGGCCCATACCGTGCGCGACCACCTCGTGGGGCGCTGGATCCGCACGCAGCAGCACTACTATGAGAAGGACC

#### 4.6.3. Gene Editing

We started from a 10 cm culture plate of the McArdle iPSC line MA1 [28] at approximately 70% confluence and treated with StemMACS™ Y27632 Rock inhibitor for about 2 h (Miltenyi Biotec, Bergisch Gladbach, Germany; #130-103-922). The gene editing was performed following the protocol previously described by Bruntraeger et al., 2019 [52]. This protocol is based on the generation of CRISPR/Cas9 RNP complexes and the use of ssODNs to drive HDR mechanisms. We used a high-fidelity Cas9 Alt-R^®^ S.p. HiFi Cas9 Nuclease V3 (IDT, Newark, NJ, USA; #1081060) to form the RNPs. That way a more precise editing and lower off-targets risk was ensured. The nucleofection was performed in a single-cell suspension of 1·10^6^ cells using the P3 Primary Cell 4D-Nucleofector^®^ X Kit L (Lonza, Basel, Switzerland; #V4XP-3012) in a 4D Nucleofector System by Lonza (Program CA-137) following the manufacturer’s instructions. Subsequently, the potentially edited iPSCs were cultured and expanded for further experiments as described above.

#### 4.6.4. Evaluation of the On-Target Efficiency: T7E1 Assay

The on-target cleavage efficiency of the Cas9 enzyme in combination with the two different sgRNAs designed was evaluated using a classic T7 endonuclease I assay. To this end, we nucleofected the cells in the presence only of the RNPs formed by the Cas9 and either sgRNA.1 or sgRNA.2, just to check which one of the designed sgRNAs displayed a higher efficiency. We extracted genomic DNA from the pool of nucleofected cells with the NucleoSpin^®^ Tissue kit (Macherey–Nagel, Düren, Germany; #740952.50) following the manufacturer’s instructions. Then we amplified the region of interest using the primers PYGM-Fw (5′-CAGAAGACCATGTGCAAGGC-3′) and PYGM-Rv (5′-CTTAAGTCAAGATCGCCAGCTC-3′) for 35 cycles with the GoTaq^®^ G2 Hot Start Polymerase (Promega, Madison, WI, USA; #M7406). The PCR products were purified from the gel using the NZYGelpure kit (NzyTech, Lisbon, Portugal; #MB01102). Subsequently, 200 ng of the purified PCR product were employed to perform the heteroduplex formation followed by a digestion with the enzyme T7 endonuclease I (New England Biolabs, Ipswich, MA, USA; #M0302S) as described by the manufacturer. The digested product was loaded in a 10% polyacrylamide gel, and run at 140 V for 90 min. We stained the gel with GreenSafe Premium (NzyTech, Lisbon, Portugal; #MB13201) for 30 min in darkness. The percentage of gene modification was estimated following the formula described by Fuster et al., 2017 [53].

#### 4.6.5. Evaluation of the Edition Efficiency: RFLP

The edition efficiency was evaluated with a Restriction Fragment Length Polymorphism (RFLP). For this purpose, we extracted the genomic DNA of the edited cells with the Cas9/sgRNA.2 RNPs and the ssODN repair template. Subsequently, we amplified and purified the region of interest as described in the previous section. Using 500 ng of the purified product we performed the digestion with the enzyme *XhoI* (New England Biolabs, Ipswich, MA, USA; #R0146S), following the manufacturer’s instructions. We loaded the product in a 2% agarose gel, stained with GreenSafe Premium. The percentage of editing was calculated taking into account the band intensities of the cleaved fractions, using the ImageJ software (NIH).

#### 4.6.6. Subcloning

After reaching approximately 70% confluence, the pool of edited iPSCs was detached using Accutase™ (StemCell Technologies, Vancouver, Canada; #07920) to create a single-cell suspension. We seeded 1000 cells on hESC-qualified Matrigel-coated 10 cm plates with cloning medium, composed of mTeSR™1 and CloneR™ (StemCell Technologies, Vancouver, Canada; #05888), and we left the plates undisturbed for 48 h. After that, we changed the cloning medium as described by the manufacturer. The culture was maintained until the colonies had an approximate 1–2 mm diameter.

#### 4.6.7. Freezing and Analysis of the Clones

We manually picked round shaped and good-looking colonies, discarding the ones that were uncertain to come from a single cell. We picked a total of 96 colonies, which were split into two different wells from two separate hESC-qualifed Matrigel-coated 96-well plates to create replicates. When they reached an appropriate size, we froze the colonies from one of the 96-well plates in Gibco™ KnockOut™ Serum Replacement (Gibco, Waltham, MA, USA; #10828010) with 10% final DMSO. The colonies from the duplicate 96-well plate were lysate using Yolk Sac lysis buffer (10 mM Tris–HCl pH 8.3, 50 mM KCl, 2 mM MgCl2, 0.45% IGEPAL CA-630, 0.45% Tween 20) supplemented with Proteinase K (GE Healthcare, Chicago, IL, USA; #406172). We incubated the plate at 60 °C for 1 h, followed by an inactivation at 95 °C for 10 min. We then performed a PCR with a 1:10 dilution of the lysates using the same conditions described above. The PCR products were purified using the MultiScreen PCRμ_96_ filtration plates (Millipore, Burlington, MA, USA; #LSKMPCR10) and sequenced by Sanger to assess the edition. One of the successfully edited clones (labelled as clone B9) was selected for further experiments. This clone was thawed from the frozen 96-well backup plate and expanded as described above. The correct edition of the mutation was double-checked by Sanger sequencing.

#### 4.6.8. Off-Targets Analysis

We selected six different possible off-targets from the most likely ones (with 3–4 mismatches and affecting either a coding region or an RNA gene) predicted by both IDT and CRISPOR design tools to check if the CRISPR/Cas9 system had modified their sequences. We amplified each region by PCR using the primers listed in Table 7. Subsequently, we purified the PCR products as described above and sequenced them by Sanger.

#### 4.6.9. Pluripotency and Integrity Assessment

To verify if MA1-B9 was a bona fide iPSC line, a complete battery of tests was performed to evaluate its integrity and pluripotency after the edition. The pluripotency of the lines was evaluated by immunofluorescence, RT-qPCR and in vitro differentiation. The quality of the line was demonstrated by STR analysis, mycoplasma detection and karyotype analysis. All the protocols are detailed in Section 4.2.

### 4.7. Pilot Study to Evaluate Two Read-Through Compounds in the Isogenic McArdle Disease Model

The line MA1 and its matched isogenic control MA1-B9 were differentiated towards innervated skeletal muscle following the protocol described in Section 4.4. The expression of some myogenic markers (*PAX3*, *PAX7*, *TTN*, *DES* and *MYOD1*) and a motor neuron marker (*ChAT*) were assessed by RT-qPCR at day 40 of differentiation, following the protocol detailed in Section 4.4.1.

Both cell lines were treated, between days 41 and 43 of differentiation, with two read-through compounds to evaluate the possible rescue of the *PYGM* expression. The drugs tested were the following:

- PTC124 (Merck, Darmstadt, Germany; #5309180001). Treatment at 2, 5, 10 and 50 μM.
- RTC13 (Merck, Darmstadt, Germany; #SML1725). Treatment at 2, 5, 10 and 50 μM.

Cell pellets were collected after 24 h of treatment with both drugs. Additionally, the possible effect of caffeine (Merck, Darmstadt, Germany; #C0750) to enhance the action of PTC124 [45] was also evaluated. To do so, cells were treated with 0.5 mM caffeine along with the previously described concentrations of PTC124, collecting cell pellets after 24 h. Subsequently, the effect of the different drugs on the expression of *PYGM* was compared by RT-qPCR. For this purpose, total RNA was extracted from cell pellets using a classic protocol with TRI^®^ reagent (Thermo Fisher Scientific, Waltham, MA, USA; #AM9738), and 1 μg of RNA was retrotranscribed using the RevertAid RT Reverse Transcription Kit (Thermo Fisher Scientific, Waltham, MA, USA; #K1691), according to the manufacturer’s instructions. PYGM expression analysis was performed with a multiplex gene expression assay using TaqMan™ probes, as described in Section 4.5.1. The values are representative of, at least, three independent replicates. Statistical analyses were performed with a Sidak or Dunnet’s multiple comparisons two-way ANOVA tests, considering as statistically significant a *p*-value < 0.05.

## 5. Conclusions

We have established the first human in vitro model for McArdle disease based on iPSC technology. This model can constitute the perfect platform to perform high-throughput drug screenings or drug repurposing studies, which could help to identify a therapy for McArdle disease.

## Figures and Tables

**Table 1:** Primary and secondary antibodies to evaluate the expression of pluripotency markers by immunocytochemistry.

Primary Antibodies		
Name	Dilution	Reference
Goat anti-NANOG	1:25	R&D Systems, Minneapolis, MN, USA; #sc-5279
Mouse anti-OCT4	1:100	Santa Cruz Biotechnology, Dallas, TX, USA; #sc-5279
Rabbit anti-SOX2	1:100	Thermo Fisher Scientific, Waltham, MA, USA; #PA1-16968
Mouse anti-SSEA4	1:10	Millipore, Burlington, MA, USA; #MAB4304
Rat anti-SSEA3	1:20	Abcam, Cambridge, UK; #ab16286
Mouse anti-TRA-1-81	1:150	Millipore, Burlington, MA, USA; #MAB4381
Mouse anti-TRA-1-60	1:150	Millipore, Burlington, MA, USA; #MAB4360
**Secondary antibodies**		
**Name**	**Dilution**	**Reference**
Cy™2-conjugated AffiniPure Donkey Anti-Goat IgG (H + L)	1:50	Jackson ImmunoResearch Labs, Ely, UK; #705-225-147
Cy™2-conjugated AffiniPure Goat Anti-Mouse IgG, Fcγ subclass 2b specific	1:50	Jackson ImmunoResearch Labs, Ely, UK; #115-225-207
Cy™3-conjugated AffiniPure Donkey Anti-Mouse IgM, μ chain specific	1:250	Jackson ImmunoResearch Labs, Ely, UK; #715-165-020
Cy™2-conjugated AffiniPure Goat Anti-Rabbit IgG (H + L)	1:50	Jackson ImmunoResearch Labs, Ely, UK; #111-225-144
Cy™3-conjugated AffiniPure Goat Anti-Rat IgM, μ chain specific	1:250	Jackson ImmunoResearch Labs, Ely, UK; #112-165-075
Cy™3-conjugated AffiniPure Goat Anti-Mouse IgG, Fcγ subclass 3 specific	1:250	Jackson ImmunoResearch Labs, Ely, UK; #115-165-209

**Table 2:** Primary and secondary antibodies used for the immunocytochemistry analysis of the markers for ectoderm (Tuj1), endoderm (AFP) and mesoderm (SMA) after the in vitro spontaneous differentiation assay.

Primary Antibodies		
Name	Dilution	Reference
Mouse anti-β tubulin isotype III (ectoderm)	1:300	Merck, Darmstadt, Germany; #T8660
Mouse anti-AFP (endoderm)	1:300	Merck, Darmstadt, Germany; #WH0000174M1
Mouse anti-SMA (mesoderm)	1:400	Merck, Darmstadt, Germany; #A2547
**Secondary antibodies**		
**Name**	**Dilution**	**Reference**
Goat anti-mouse IgG (H + L), Alexa Fluor 488	1:500	Thermo Fisher Scientific, Waltham, MA, USA #A-11029

**Table 3:** Compounds to be added to the basal medium employed in the distinct stages of the differentiation protocol from iPSCs towards innervated myogenic cells.

Differentiation Days (D)	Supplements to Be Added
From D0 to D5	Insulin-transferrin-selenium (ITS) 1x (Gibco, Waltham, MA, USA; 41400045); LDN193189 0.5 μM (StemCell Technologies, Vancouver, Canada; 72147); CHIR99021 3 μM (StemCell Technologies, Vancouver, Canada; 72054) *D1: additional supplementation with 2 μM Thiazovivin
From D6 to D7	IGF-I 4 ng/mL (StemCell Technologies, Vancouver, Canada; 78022.1); HGF 10 ng/mL (StemCell Technologies, Vancouver, Canada; 78019.1); LDN193189 0,5 μM (StemCell Technologies, Vancouver, Canada; 72147); β-mercaptoethanol 100 μM (Gibco, Waltham, MA, USA; 21985023)
From D8 to D11	IGF-I 4 ng/mL (StemCell Technologies, Vancouver, Canada; 78022.1); β-mercaptoethanol 100 μM (Gibco, Waltham, MA, USA; 21985023)
From D1 to D16	IGF-I 4 ng/mL (StemCell Technologies, Vancouver, Canada; 78022.1); β-mercaptoethanol 100 μM (Gibco, Waltham, MA, USA; 21985023); DAPT 10 μM (Merck, Darmstadt, Germany; D5942)
From D17-onwards	IGF-I 4 ng/mL (StemCell Technologies, Vancouver, Canada; 78022.1); β-mercaptoethanol 100 μM (Gibco, Waltham, MA, USA; 21985023)

**Table 4:** Primary and secondary antibodies used to assess the expression of myogenic and motor neuron proteins.

Primary Antibodies		
Name	Dilution	Reference
Mouse anti-titin	96:1000	DSHB, Iowa, IA, USA; #9D10
Rabbit anti-desmin	1:100	Abcam, Cambridge, UK; #AB15200
Chicken anti-neurofilament	1:2000	Biolegend, San Diego, CA; USA; #PCK-593P
Rabbit anti-MNX1 (HB9)	1:100	Millipore, Burlington, MA, USA; #ABN174
Goat anti-ChAT	1:100	Millipore, Burlington, MA, USA; #AB144P
**Secondary antibodies**		
**Name**	**Dilution**	**Reference**
Goat anti-mouse IgG (H + L) Alexa Fluor^®^ 647	1:1000	Thermo Fisher Scientific, Waltham, MA, USA; #A-21236
Goat anti-chicken IgG (H + L) DyLight™ 488	1:1000	Rockland, Pottstown, PA, USA; #603-141-126
Goat anti-rabbit IgG (H + L) Alexa Fluor^®^ 594	1:1000	Thermo Fisher Scientific, Waltham, MA, USA; #A-11012
Goat anti-mouse IgG (H + L) Alexa Fluor^®^ 488	1:1000	Thermo Fisher Scientific, Waltham, MA, USA; #A28175
Goat anti-rabbit IgG (H + L) Alexa Fluor^®^ 555	1:1000	Cell Signalling, Danvers, MA, USA; #4413
Donkey anti-goat IgG (H + L) Alexa Fluor^®^ 633	1:1000	Thermo Fisher Scientific, Waltham, MA, USA; #A-21082

**Table 5:** Primers for the evaluation of the expression of several myogenic and motor neuron genes by RT-qPCR.

Target	Forward Primer (5′→3′)	Reverse Primer (5′→3′)
*MyH2*	GGAGCTGGTGGAGGGGCCAA	TGCTCCATGGCACCAGGAGTTT
*MyH3*	GCTTGTGGGCGGAGGTCTGG	AGGGCTGGTTCTGAGCCTCGAT
*MyoD*	TGCGCAACGCCATCCGCTA	GGGCCGCTGTAGTCCATCATGC
*TTN*	CCGAAATGCATCAGTCAGCG	CCTTGCAAGCTTGTGTCACC
*DES*	CCGCCATCTGCGCGAGTACC	TGCTCAGGGCTGGTTTCTCGGA
*PAX3*	CACCAGGCATGGATTTTCC	TTGTCAGGAGTCCCATTACCT
*PAX7*	CCACAGCTTCTGCAGCTACTCTG	GGGTTGCCCAAGATGCTG
*ChAT*	AGAAGCAGAAATGCAGCCCT	GCTCTCACAAAAGCCAGTGC
*HPRT*	CATTATGCTGAGGATTTGGAAAGG	CTTGAGCACACAGAGGGCTACA
*PPIA*	GGCAAATGCTGGACCCAACACA	TGCTGGTCTTGCCATTCCTGGA

**Table 6:** Sequence of the crRNA domain and PAM of the two sgRNAs designed.

	crRNA Domain Sequence (5′ → 3′)	PAM
sgRNA.1	ACTCGTAAAGGACCGCAATG	TGG
sgRNA.2	GAGCAAAGTAGTAGTCTCAT	GGG

**Table 7:** Primers designed for the PCR amplification of the selected off-target regions.

Target	Forward Primer (5′ → 3′)	Reverse Primer (5′ → 3′)
IL17RD	CCACCTCAACAGAGACCACC	GGGGGCCAGAGAGTTTTCTT
CMPK1	GCATTCCTACTCACATAAGTG	GTCATATTATCTCAATCAACTC
SLC39	TTCTCTAGATATACTCAGCC	TATAAGGCAGCCATCCATG
LOC105375144	GCTGGTAGCACAAGCAGAG	AGTCCAGTGTGATAGGAGCC
XPO4	CAGCATTGGGCAGATTACTTCTT	GGGGACAGAATAGTTTCATAGGCA
RIT1	AGGGACCACTACTCAGAGCT	AGACATCAGGGGTGTGGGTA

## Data Availability

Data is contained within the article or supplementary material.

## Supplementary Materials

The supporting information can be downloaded at: https://www.mdpi.com/article/10.3390/ijms232213964/s1.
