# Supplementary material for: Generation of the First Human In Vitro Model for McArdle Disease Based on iPSC Technology
Source: Int J Mol Sci. 2022 Nov 12;23(22):13964. doi: 10.3390/ijms232213964 (PMC9692531; doi:10.3390/ijms232213964)
Supplement: Supplementary file 1 [file ijms-23-13964-s001.zip › Supplementary material.pdf]

Supplementary Figures

Generation of the First Human In Vitro Model for McArdle Disease Based on iPSC Technology

María del Carmen Ortuño-Costela [1,2], Victoria Cerrada [1], Ana Moreno-Izquierdo [3], Inés García-Consuegra [4,5], Camille Laberthonnière [6], Mégane Delourme [6], Rafael Garesse [2], Joaquín Arenas [4,5], Carla Fuster García [5,7], Gema García García [5,7], José María Millán [5,7], Frédérique Magdinier [6] and María Esther Gallardo [1,*]

Figure S1. Assessment of pluripotency and integrity of the iPSC line C10. (a) Alkaline phosphatase positive staining. (b) Immunofluorescence analysis showing the positive expression of the pluripotency nuclear markers NANOG, OCT4 and SOX2, and the surface markers SSEA3, SSEA4, TRA-1-81 and TRA-1-60. (c) RT-qPCR to evaluate the expression levels of different pluripotency genes: *OCT4, SOX2, CRIPTO, NANOG* and *REX1*. We can confirm the increase in the expression of these genes compared to the starting fibroblasts. The values represent the expression mean of at least three replicates, and they are relative to the expression of *GAPDH* as housekeeping gene. Error bars show standard deviation. (d) *In vitro* differentiation assay to confirm the capacity of the iPSC line C10 to differentiate towards cell types of the three germ layers: ectoderm (positive for β-III-tubulin, Tuj1), mesoderm (positive for smooth muscle actin, SMA), and endoderm (positive for α-fetoprotein, AFP). (e) Mycoplasma test for the iPSC line C10, along with a control with water as sample (H_2O), a mycoplasma-free negative control (C-), and a sample positive for mycoplasma (C+). The presence of a band at 300 bp confirms a sample to be mycoplasma-positive. (f) Assessment of the Sendai virus silencing by RT-PCR after the reprogramming process. A negative control without the vectors (C-), a positive Sendai virus control (C+) and the original fibroblasts as comparative sample were included. (g) Normal 46, XY karyotype of the iPSC line C10, analysed by G banding.

Figure S2. Assessment of pluripotency and integrity of the iPSC line MA4. (a) Sanger sequencing to confirm the mutation *PYGM* c.148C>T; p.R50* in the iPSC line MA4, present as well in the original fibroblasts but not in a control cell line. (b) Alkaline phosphatase positive staining. (c) RT-qPCR to evaluate the expression levels of different pluripotency genes: *OCT4, SOX2, CRIPTO, NANOG* and *REX1*. We can confirm the increase in the expression of these genes compared to the starting fibroblasts. The values represent the expression mean of at least three replicates, and they are relative to the expression of *GAPDH* as housekeeping gene. Error bars show standard deviation. (d) Immunofluorescence analysis showing the positive expression of the pluripotency nuclear markers NANOG, OCT4 and SOX2, and the surface markers SSEA3, SSEA4, TRA-1-81 and TRA-1-60. (e) *In vitro* differentiation assay to confirm the capacity of the iPSC line MA4 to differentiate to cell types of the three germ layers: ectoderm (positive for β-III-tubulin, Tuj1), mesoderm (positive for smooth muscle actin, SMA), and endoderm (positive for α-fetoprotein, AFP). (f) Normal 46, XX karyotype of the iPSC line MA4, analysed by G banding. (g) Assessment of the Sendai virus silencing by RT-PCR after the reprogramming process. A negative control without the vectors (C-), a positive Sendai virus control (C+) and the starting McArdle fibroblasts as comparative sample were included. (h) Mycoplasma test for the iPSC line MA4, along with a control with water as sample (H_2O), a negative control known to be mycoplasma-free (C-), and a mycoplasma-positive sample (C+). The presence of a band at 300 bp confirms a sample to be mycoplasma-positive.

Figure S3. Pluripotency and integrity analysis of the MA1-B9 line. (a) Immunofluorescence assay showing the positive expression of the pluripotency nuclear markers NANOG and OCT4, along with the surface marker TRA-1-81. Scale bar: 50 µm. (b) RT-qPCR assay to check the levels of expression of several pluripotency genes (*OCT4*, *SOX2*, *CRIPTO*, *NANOG* and *REX1*) in the edited line MA1-B9 compared to human embryonic stem cells (hESC) and to the IISHDOi007-A iPSC line (previously generated in our laboratory). Values represent expression mean of at least three independent replicates, relative to the expression of *GAPDH* as housekeeping gene. Error bars show standard deviation. (c) *In vitro* differentiation assay to validate the capacity of the MA1-B9 line to generate cell types of the three germ layers: mesoderm (positive for SMA), endoderm (positive for AFP), and ectoderm (positive for Tuj1). Scale bar: 50 µm. (d) Normal 46, XX karyotype of the edited iPSC line MA1-B9. (e) Mycoplasma detection in the edited line MA1-B9 (labelled here as B9). The absence of band at 300 bp shows that the line is mycoplasma free. C-: negative control, water as sample; C+: positive control, mycoplasma-positive sample.

Figure S1

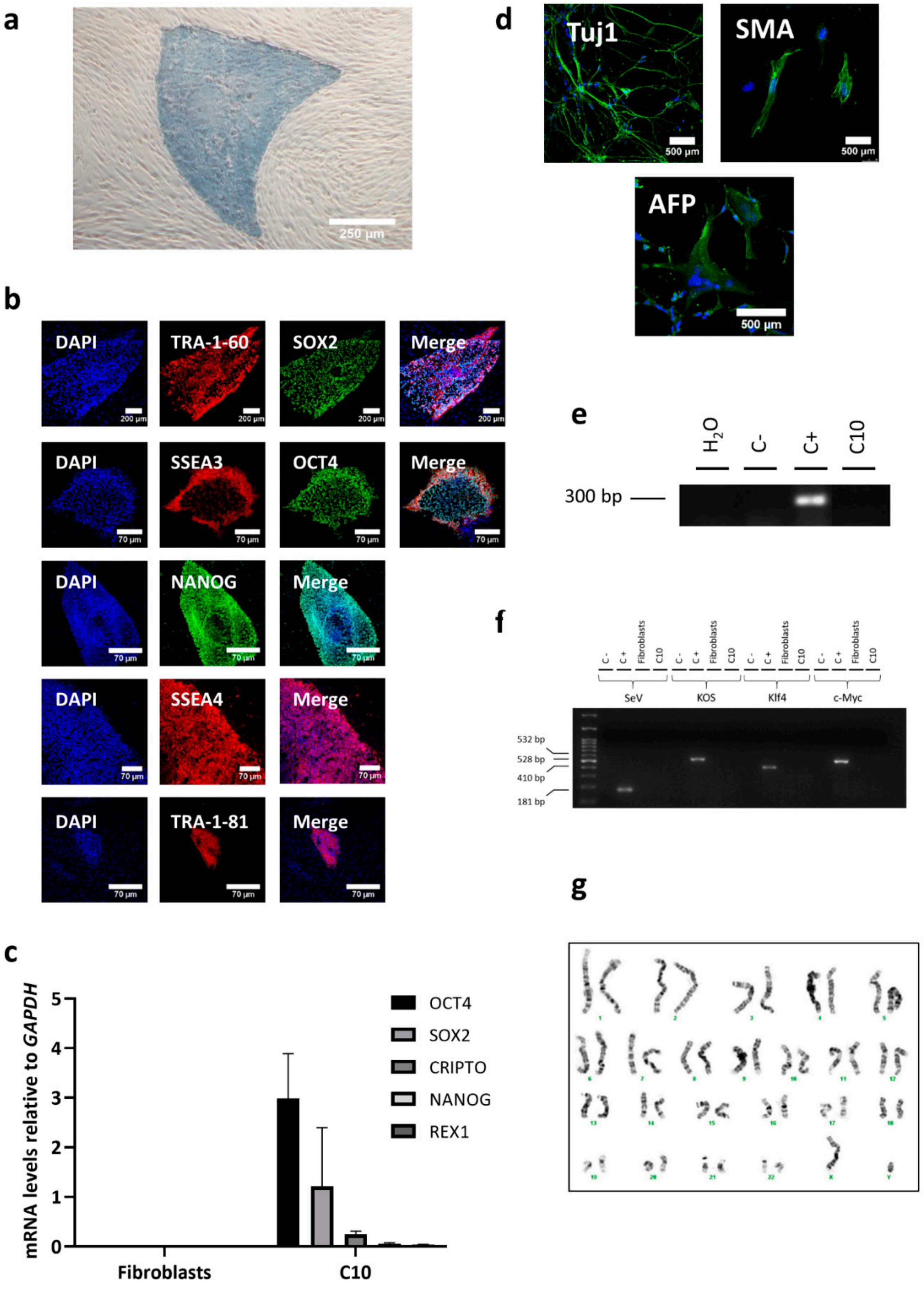

a
250 µm
b
DAPI
TRA-1-60
SOX2
Merge
200 µm
SSEA3
OCT4
70 µm
NANOG
SSEA4
TRA-1-81
c
mRNA levels relative to GAPDH
0
1
2
3
4
5
Fibroblasts
C10
OCT4
SOX2
CRIPTO
NANOG
REX1
d
Tuj1
SMA
AFP
500 µm
e
H2O
C-
C+
C10
300 bp
f
C-
C+
Fibroblasts
C10
SeV
KOS
Klf4
c-Myc
532 bp
528 bp
410 bp
181 bp
g

Figure S2

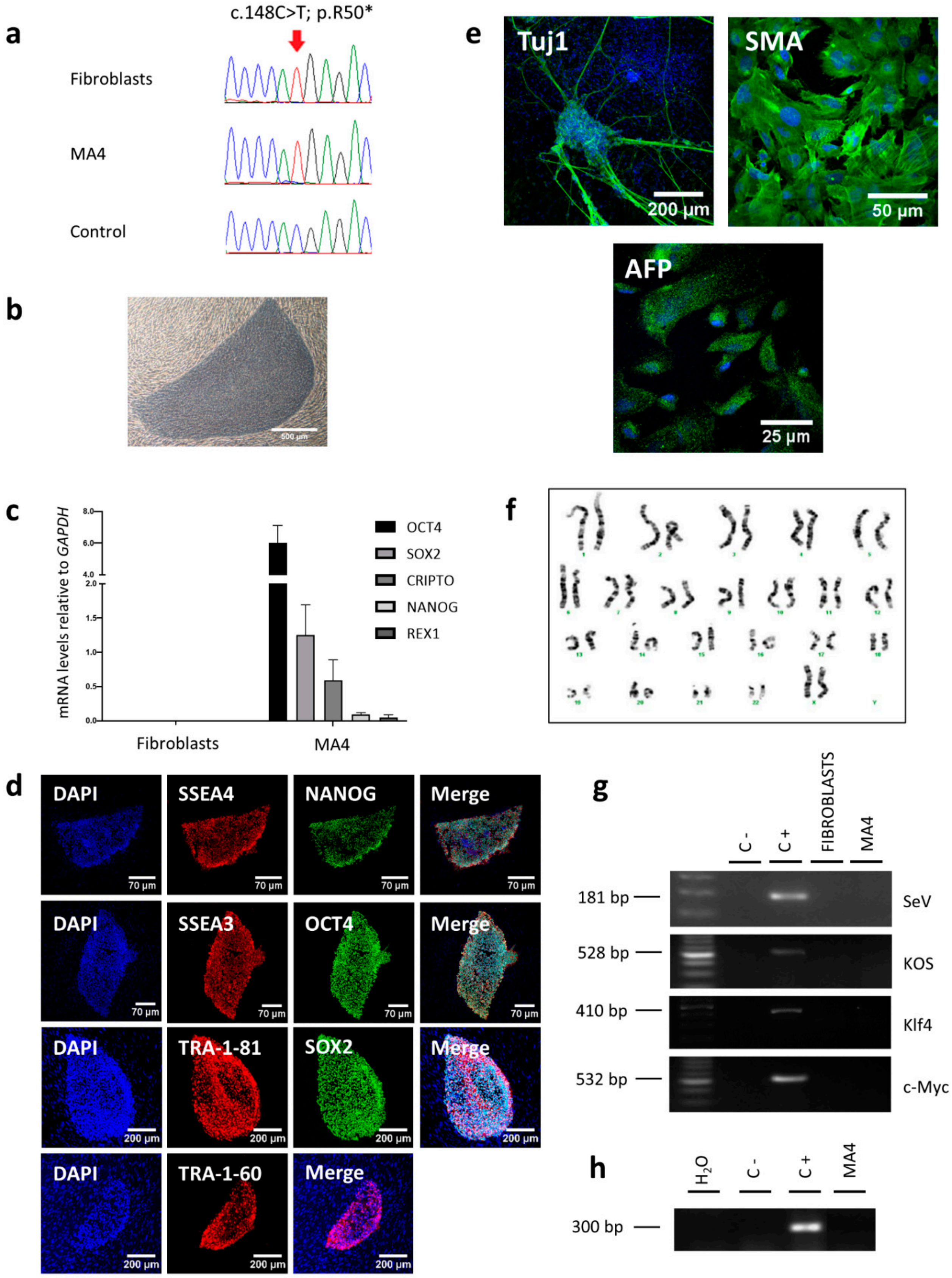

a
c.148C>T; p.R50*
Fibroblasts
MA4
Control
b
c
mRNA levels relative to GAPDH
OCT4
SOX2
CRIPTO
NANOG
REX1
Fibroblasts
MA4
d
DAPI
SSEA4
NANOG
Merge
SSEA3
OCT4
TRA-1-81
SOX2
TRA-1-60
70 µm
200 µm
e
Tuj1
SMA
AFP
200 µm
50 µm
25 µm
f
g
C -
C +
FIBROBLASTS
MA4
181 bp
528 bp
410 bp
532 bp
SeV
KOS
Klf4
c-Myc
h
H2O
300 bp

Figure S3

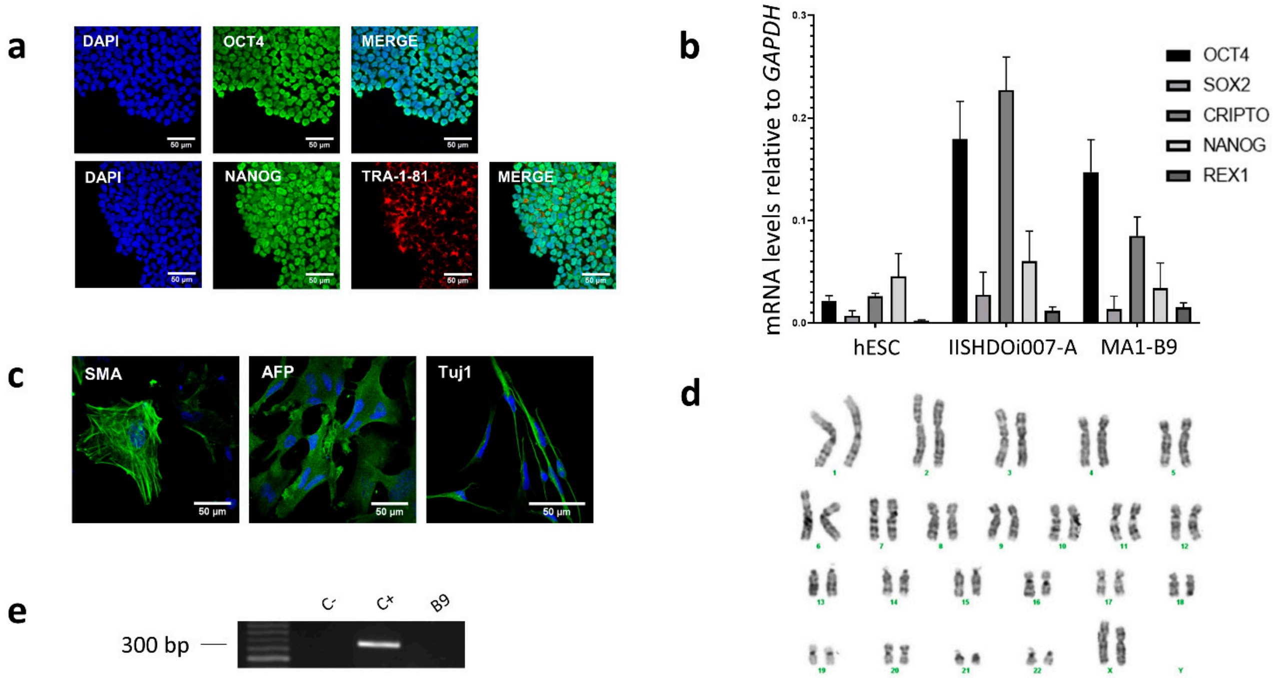
a
DAPI
OCT4
MERGE
DAPI
NANOG
TRA-1-81
MERGE
b
mRNA levels relative to GAPDH
0.3
0.2
0.1
0.0
OCT4
SOX2
CRIPTO
NANOG
REX1
hESC
IISHDOi007-A
MA1-B9
c
SMA
AFP
Tuj1
50 µm
d
e
300 bp
C-
C+
B9
